# A pilot study examining the effects of low-volume high-intensity interval training and continuous low to moderate intensity training on quality of life, functional capacity and cardiovascular risk factors in cancer survivors

**DOI:** 10.7717/peerj.2613

**Published:** 2016-10-20

**Authors:** Kellie Toohey, Kate L. Pumpa, Leonard Arnolda, Julie Cooke, Desmond Yip, Paul S. Craft, Stuart Semple

**Affiliations:** 1 Discipline of Sport and Exercise Science/Faculty of Health, University of Canberra, Canberra, ACT, Australia; 2 Research Institute for Sport and Exercise, University of Canberra, Canberra, ACT, Australia; 3 ANU Medical School, Australian National University, Canberra, ACT, Australia

**Keywords:** Exercise, High-intensity, Moderate-intensity, Cancer survivors, Cardiovascular disease, Health, Functional capacity, Quality of life, Training, Physical activity

## Abstract

**Purpose:**

The aim of this study was to evaluate the effects of low-volume high-intensity interval training and continuous low to moderate intensity training on quality of life, functional capacity and cardiovascular disease risk factors in cancer survivors.

**Methods:**

Cancer survivors within 24 months post-diagnosis were randomly assigned into the low-volume high-intensity interval training group (*n* = 8) or the continuous low to moderate intensity training group (*n* = 8) group for 36 sessions (12 weeks) of supervised exercise. The low-volume high-intensity interval training (LVHIIT) group performed 7 × 30 s intervals (≥85% maximal heart rate) and the continuous low to moderate intensity training (CLMIT) group performed continuous aerobic training for 20 min (≤55% maximal heart rate) on a stationary bike or treadmill.

**Results:**

Significant improvements (time) were observed for 13 of the 23 dependent variables (ES 0.05–0.61, *p* ≤ 0.05). An interaction effect was observed for six minute walk test (18.53% [32.43–4.63] ES 0.50, *p* ≤ 0.01) with the LVHIIT group demonstrating greater improvements.

**Conclusion:**

These preliminary findings suggest that both interventions can induce improvements in quality of life, functional capacity and selected cardiovascular disease risk factors. The LVHIIT program was well tolerated by the participants and our results suggest that LVHIIT is the preferred modality to improve fitness (6MWT); it remains to be seen which intervention elicits the most clinically relevant outcomes for patients. A larger sample size with a control group is required to confirm the significance of these findings.

## Introduction

There is strong evidence indicating that cancer survivors may be at an increased risk for developing cardiovascular disease (CVD) (Hooning et al., 2007; Darby et al., 2013). This may be as a result of cancer related therapy and changes in lifestyle patterns after diagnosis (Hooning et al., 2007). Whilst it is well established that exercise reduces CVD risk in an apparently healthy population (O’Donovan et al., 2010; Dunkley et al., 2012; Perk et al., 2012), it is unclear if the same is true for cancer survivors.

The benefits of low to moderate intensity exercise for cancer survivors have been well reported and is deemed to promote improvements in aerobic fitness, functional capacity and psychological factors (Mutrie et al., 2007; McNeely et al., 2006). Continuous moderate intensity training (CMIT) is commonly prescribed in the usual care for cancer survivors but there is a growing body of evidence that high-intensity interval training (HIIT) may be one of the most effective ways of improving cardiometabolic health (Kessler, Sisson & Short, 2012; Weston et al., 2014). High-intensity interval training (HIIT) is not commonly prescribed in clinical practice for cancer survivors, however, its application as a modality for the treatment and management of chronic disease is gathering momentum and research studies in chronic disease populations such as; cardiac (Aamot et al., 2014) stroke (Mattlage et al., 2013), diabetes (Gillen et al., 2012), cancer (Gibala et al., 2012), and hypertension (Brito et al., 2014) have recently been published. The growing body of evidence suggests that HIIT may impart benefits that are greater than those elicited through low or moderate intensity training in healthy populations, but less has been published on cancer survivors (Adamsen et al., 2009).

A systematic review and meta-analysis conducted by Gist et al. in 2013 reported that using HIIT was as effective as CMIT in improving cardiorespiratory fitness in healthy people, but with a reduction in volume of activity (Gist et al., 2014). Low-volume high-intensity interval training has been shown to elicit positive changes in the sedentary population by improving VO_2_ peak and insulin sensitivity (Metcalfe et al., 2012), and it is plausible that it could be an alternative and time efficient training mode for sedentary cancer survivors.

HIIT is characterised by brief repeated high-intensity work efforts, interspersed with periods of rest or lower intensity exercise during recovery (Metcalfe et al., 2012). HIIT has been shown to be an effective alternative to endurance training for inducing cardiovascular improvements and metabolic adaptations within skeletal muscle, improving aerobic exercise performance and VO_2max_ (Metcalfe et al., 2012; Sloth et al., 2013; Kodama et al., 2009). This method of training has also been shown to increase mitochondrial content in a similar way to endurance training, but with improved efficiency, markedly lower total exercise volume and time commitment (Burgomaster et al., 2008). Gibala et al. (2012) reports that just ten minutes per week of HIIT produced the same changes in muscle metabolic control and cardiovascular function when compared to four and a half hours per week of CMIT. Similar cardiovascular adaptations may occur with HIIT when compared with CMIT, but with less work and time commitment overall (Wisloff, Ellingsen & Kemi, 2009; Tjonna et al., 2009). These potential benefits have not been thoroughly explored in cancer survivors and it is clear that we have yet to define what the optimal dose, frequency and intensity of exercise should be to maximise health benefits for cancer survivors. Therefore, the purpose of this pilot project was to examine the effects of LVHIIT (low volume high intensity interval training) versus continuous low to moderate intensity training (CLMIT) on CVD risk, and other health related outcomes, in cancer survivors.

## Methods

### Participants

Twenty four cancer survivors were recruited for this study via pamphlet distribution, online social media and by word of mouth. The period of recruitment was from February–September 2014. Referrals were also obtained from general practitioners, oncologists, nurses and community organisations that support cancer survivors. Inclusion criteria were; participants within the first 24 months of diagnosis, and the post treatment phase of the physical activity across the cancer experience (PEACE) organizational model, once the acute effects of medical treatments had dissipated (Courneya & framework, 2001); participation in less than 30 min of moderate intensity aerobic physical activity five days per week or participation in less than 20 min of vigorous intensity aerobic activity three days per week, as recommended by the American College of Sports Medicine (ACSM) (Gordon, 2009). Participants were excluded if they had brain or bone metastatic cancers, bone pain, resting blood pressure >180/110 mmHg, if they were pregnant or undergoing psychotherapy or if they had any musculoskeletal injuries or disabilities restricting their ability to participate in physical activity. All participants completed a medical health screen questionnaire, provided informed consent and received physician clearance prior to participating in the program. All procedures performed in studies involving human participants were in accordance with the ethical standards of the institutional and/or national research committee and with the 1964 Helsinki declaration and its later amendments. The study was approved by the University of Canberra Human Research Ethics Committee (UCHREC13-153).

**Figure 1:**
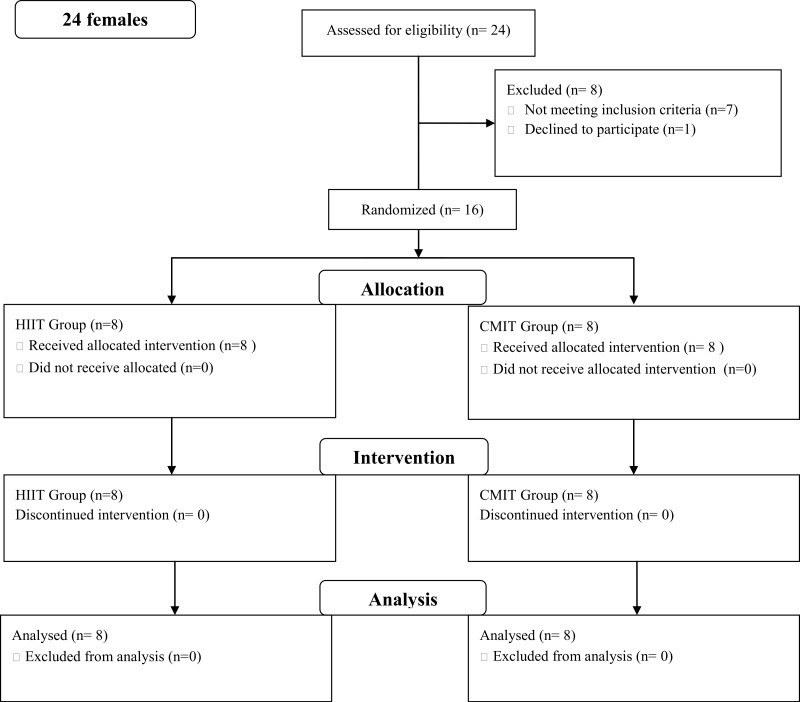
Flow diagram. Consort chart of recruitment into the study.

Twenty four females were assessed and 16 completed the study (see Fig. 1). Seven individuals did not meet the inclusion criteria and one declined to participate. Six participants were outside the 24 month post-diagnosis period and one participant was undergoing treatment. Individuals were randomly assigned into either a LVHIIT (*n* = 8) or CLMIT (*n* = 8) group (Fig. 1).

Participants mean age was 51.6 y (±13.01y). Cancer diagnosis included an independent diagnosis of colon (*n* = 1), cervical (*n* = 1), melanoma (*n* = 1), ovarian (*n* = 2), breast (*n* = 9) and a diagnosis breast and uterine (*n* = 1) and breast and liver (*n* = 1). Treatment types included; surgery (n = 3), surgery plus chemotherapy (*n* = 2), surgery plus radiation (*n* = 1), surgery plus chemotherapy plus adjuvant endocrine treatment (*n* = 2) and surgery plus chemotherapy plus radiation plus adjuvant endocrine treatment (*n* = 8).

### Quality of life

**Figure 2:**
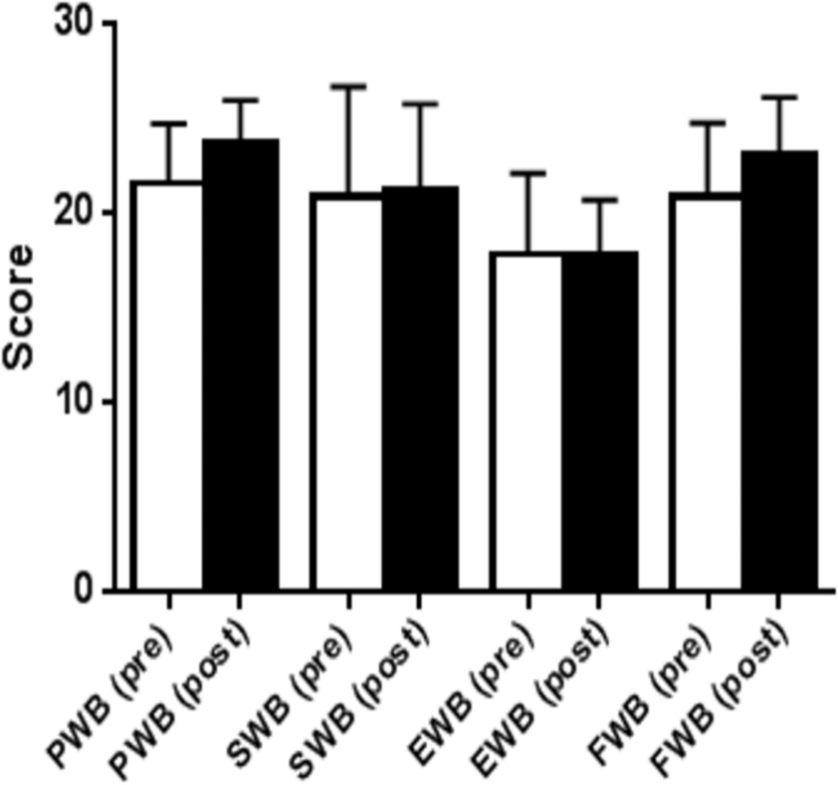
Quality of life results from FACT G in the continuous low to moderate intensity group. QoL domains, physical well-being (PWB); social well-being (SWB), emotional well-being (EWB); functional well-being, (FWB).

**Figure 3:**
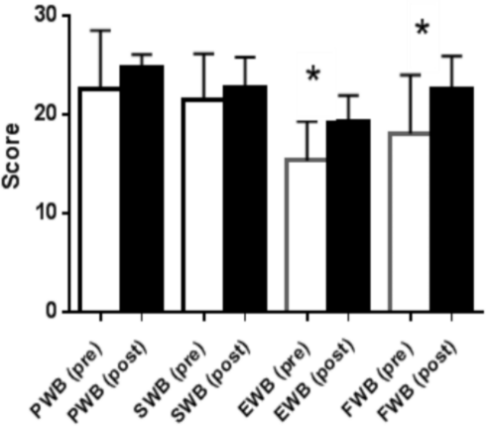
Quality of life results from FACT GQuestionnaire in the low volume high-intensity group. QoL domains, physical well-being (PWB); social well-being (SWB), emotional well-being (EWB); functional well-being, (FWB). (^∗^*p* < 0.05).

Functional capacity and quality of life (QOL) were measured using the Functional Assessment of Cancer Therapy-General (FACT–G) questionnaire (version 4) (Esper et al., 1997). The FACT-G is a 27 item compilation of general questions, divided into four QOL domains: physical well-being, social/family well-being, emotional well-being and functional well-being. This validated survey is commonly used for cancer patients (Overcash et al., 2001) (see Figs. 2 and 3).

### Anthropometrics

Total body composition was determined by a Dual X-ray Absorptiometry (DXA) scan (Lunar Prodigy Pro scanner; GE Lunar Corp., Madison, WI USA) (Bergman et al., 2011). The machine undergoes daily quality control checks and is calibrated prior to use each day, using a phantom spine and the recommended machine protocols as per the manufacturers’ guidelines. Lean mass (lean) and weight (mass) and body fat percentage (fat %) were measured using DXA. Hip and waist circumferences were measured using a standard tape measure by the same individual at both testing times.

### Cardiovascular

Measures of pulse wave velocity (PWV) and pulse wave analysis (PWA) were obtained non-invasively using the SphygmoCor XCEL system (SphygmoCoR; At-Cor Medical Pty Ltd., Sydney, Australia). PWA included measures of resting heart rate (RHR), augmentation index (stiffness) (AIx), central systolic blood pressure (CSP), central diastolic blood pressure (CDP), central pulse pressure (PP), mean arterial pressure (MAP), and systolic blood pressure (SBP) and diastolic blood pressure (DBP). Carotid-femoral PWV was measured and is the recognised gold standard non-invasive measure of aortic stiffness; a strong independent predictor of cardiovascular risk (McDonnell et al., 2013; Sharman et al., 2006). AIx was acquired by measuring arterial stiffness and pulse wave reflection. The pulse pressure (PP) waveform of the left carotid artery was measured with an applanation tonometer. All measurements were obtained with participant’s supine for 10 min. Twenty continuous waveforms were captured and at least two measurements were taken per participant with one minute rest in between measurements.

### Biomarkers

Overnight fasting blood samples were obtained from the participants and analysed for concentrations of C-reactive protein (CRP), insulin, glucose, and full blood count. The analysis was conducted by a registered off-site pathology laboratory (Capital Pathology, Canberra, Australia).

### Functional capacity

Lower body strength was assessed using a repeated sit-to-stand (STS). The participants were seated in a chair and asked to stand and sit as fast as possible five consecutive times, without the use of their arms for support  (Simmonds, 2002). The six minute walk test (6MWT) was used to determine participants’ fitness levels. Participants were asked to walk as fast as they could for six minutes and the distance traveled over the six minutes was calculated and recorded (Cote et al., 2008). The 6MWT is used with many clinical populations and is a useful measure of functional capacity; it is widely utilized in research settings for cancer survivors (Enright, 2003; Schmidt et al., 2013).

### Exercise assessment and session protocols

All training sessions were supervised by an Accredited Exercise Physiologist (AEP) and consisted of 36 sessions performed over 12 weeks (three per week). Participants were asked to not participate in exercise on the day of testing and not to consume food or caffeine within two hours prior to pre and post testing. Assessments were carried out within the seven days prior to commencement of the program and within the seven days following completion.

The exercise was carried out on either a cycle ergometer or a treadmill. The LVHIIT group performed interval training (≥85% maximal heart rate), which consisted of a five minute warm up, seven by 30s intervals, with one minute rest in between each interval, followed by a five minute cool down (adapted from Gibala et al., 2012). A gradual progression in exercise was carried out by the LVHIIT group. Individuals started the first session with three intervals and one interval was added per session over the following four sessions, by the 5th exercise session participants carried out all seven intervals. A rest period of one minute was used between each interval. The CLMIT group performed continuous aerobic training (≤55% maximal heart rate) for 20 min also with a five minute warm up and cool down. The relative intensity was determined by the calculation of 220-age, 55% of the maximal heart rate for the CLMIT group and 85% of the maximal heart rate was calculated for the LVHIIT group. The two exercise protocols were matched for energy expenditure using the calculation reported by Rognmo et al. (2004). Each participant recorded their heart rate (HR) every minute for the CLMIT sessions. The peak HR in each interval and the resting HR between each interval was recorded during the LVHIIT sessions. Blood pressure was monitored immediately pre and after a minimum of five minutes rest post exercise.

### Statistical analysis

All measured variables are presented as means (standard deviation) for baseline and post-testing. Percentage change and 95% confidence intervals from baseline to post-test were also calculated for all variables. The data was analysed using IBM Corp. Released 2015. IBM SPSS Statistics for Windows, Version 23.0. Armonk, NY: IBM Corp. An ANCOVA was completed with pre-post differences being the dependent variable and the baseline score as the covariate, and group as a factor. Magnitudes of the standardised effects were interpreted using thresholds of 0.2, 0.5 and 0.8 (Cohen, 1988). These values correspond to small, medium and large Effect Size (ES), respectively (Cohen, 1988). The effect size reported in the table represents the interaction between baseline and group. Significance was set at *p* < 0.05.

## Results

Over the 36 sessions exercising heart rate for LVHIIT group was recoded at an average of 144.76 (±10.34) beats per minute in the intervals and 118.34 (±8.70) beats per minute in recovery. The average exercising heart rate for the CLMIT group was 95.05 (± 6.24) beats per minute. Overall session compliance was 93.75% (83.33–100%). Four participants attended all 36 sessions, and two participants missed six sessions over the intervention period.

Baseline differences were accounted for in the analysis across both groups. Significant improvements (time) were observed for 13 of the 23 dependent variables (*p* ≤ 0.05, ES 0.05–0.61). An interaction effect was observed for 6MWT (CLMIT group 1.16% [−3.85–6.17], LVHIIT group 18.53% [7.01–30.06]) and a medium ES (0.50) was seen. An interaction effect was also observed for CRP, this however was due to an increase in the CLMIT group (19.44% [−1.75–40.64]) and only a small decrease in the LVHIIT group (−5.95% [−43.18–31.28]). Full results are reported in Table 1.

**Table 1:** Changes in dependent variables at pre and post exercise intervention for continuous moderate intensity group.

Continuous low-moderate intensity training group				Low-volume high-intensity training group			Between group comparisons
Variable	Pre (*n* = 8) SD	Post (*n* = 8) SD	% change (95% CI)	Pre (*n* = 8) SD	Post (*n* = 8) SD	% change (95% CI)	Partial Eta Squared
Hip (cm)	104.69 ± 10.40	101.63 ± 9.43	−2.75% (−6.30–0.80)	110.45 ±10.94	104.38 ± 8.45	−5.22% (−9.01–1.44)	0.11^d^
Waist (cm)	90.31 ± 12.66	87.69 ± 10.15	−2.62% (−4.82–0.42)	93.5 ± 13.03	86.95 ± 9.78	−6.64% (−9.92–3.36)	0.32^d^
RHR (BPM)	75.63 ± 11.82	71.75 ± 8.51	−3.54% (−14.22–7.14)	74.75 ± 16.02	73.13 ± 14.81	−1.07% (−18.46–20.60)	0.20^d^
SBP (mmHg)	127.5 ± 8.77	126.50 ± 7.75	−0.39% (−6.70–5.93)	^b^129.86 ± 10.85	^b^122.14 ± 10.81	−5.78% (−9.94- -2.63)	0.46^d^
DBP (mmHg)	78.5 ± 10.21	83.25 ± 4.43	7.90% (−4.26–20.06)	84.5 ± 10.50	78.75 ± 71.78	−6.12% (−13.63–1.39)	0.67^d^
STS (5) (s)	9.32 ± 3.07	8.67 ± 2.81	−6.39% (−13.18–0.40)	10.23 ± 2.97	7.74 ± 2.31	−23.46% (−32.31–14.61)	0.06^d^
6MWT (m)	520.88 ± 74.25	530.56 ± 107.81	1.16% (−3.85–6.17)	502.81 ± 148.53	577.25 ± 102.55	18.53% (7.01–30.06)	0.50^c^
Mass (kg)	65.15± 8.69	65.14 ± 8.96	−0.06% (−1.03–0.91)	75.83 ± 11.58	74.01 ± 11.55	−2.43% (−4.08–0.78)	0.01
Fat %	42.11 ± 6.62	41.34 ± 7.80	−2.20% (−7.61–3.22)	43.28 ± 8.10	43.23 ± 6.79	−0.88% (−6.97–8.73)	0.03
Fat (kg)	26.31 ± 7.32	26.38 ± 7.61	0.29% (−3.71–4.29)	32.17 ± 9.55	30.57± 9.63	−5.50% (−9.61–1.39)	0.004
Lean (kg)	36.55 ± 4.80	36.49 ± 4.10	−0.09% (−1.96–2.15)	40.90 ± 5.27	40.88 ± 4.90	0.09% (−2.30–2.49)	0.22^d^
FBGL (mmol/L)	4.99 ± .50	4.93 ± .74	−1.41% (−8.12–5.29)	4.85 ± 0.22	4.73 ± 0.49	−2.40% (−10.18–5.39)	0.01
CRP (mg/L)	^a^1.5 ± 084	^a^2.00 ± 1.67	19.44% (−1.75–40.64)	^b^2.14 ± 1.21	^b^1.71 ± 0.95	−5.95% (−43.18–31.28)	0.51
Insulin (mU/L)	^b^8.76 ± 3.07	^b^8.29 ± 3.07	−0.98% (−24.12–26.07)	^a^8.30 ± 0.87	^a^6.32 ± 1.11	−24.09% (−30.52–17.65)	0.28
WBC (x10 9/L)	^b^5.09 ± 1.95	^b^5.26 ± 1.59	1.05% (−2.69–4.79)	7.30 ± 3.07	7.59 ± 3.00	5.23% (−1.64–12.10)	0.23
PWV (m/s)	6.51 ± 1.84	6.24 ± 1.11	−8.03% (−30.43–46.50)	^a^6.1625 ± 1.95	^a^6.46 ± 1.34	8.93% (−14.51–32.37)	0.55^d^
MAP (mmHg)	101.50 ± 10.35	99.13 ± 6.45	−1.51% (−9.80–6.78)	102.25 ± 15.69	92.50 ± 8.19	−8.59% (−14.61–2.56)	0.58^d^
CSP (mmHg)	123.38 ± 13.43	117.5 ± 8.83	−3.80% (−12.98–5.38)	128.37 ± 25.13	111.13 ± 4.41	−12.29% (−17.89–6.68)	0.61^d^
PP (mmHg)	35.63 ± 8.11	32.88 ± 5.69	−5.31% (−19.00–8.38)	42.38 ± 17.07	32.00 ± 7.29	−20.34% (−29.98–10.70)	0.52^d^
AP (mmHg)	8.50 ± 5.26	6.00 ± 5.26	−4.06% (−57.66–49.53)	10.38 ± 8.94	6.25 ± 5.65	−97.63% (−215.63–21.50)	0.10^d^
AIx (%)	22.13 ± 12.56	17.00 ± 15.65	−124.12% (−124.69–372.93)	23.25 ± 14.71	17.38 ± 14.74	−124.36% (−319.66–70.95)	0.05
CDP (mmHg)	86.88 ± 3.05	84.63 ± 4.31	−1.72% (−9.74–6.30)	87.38 ± 11.72	79.13 ± 8.17	−8.76% (−14.93–2.58)	0.61^d^
FACTG	81.25 ± 9.45	85.88 ± 7.38	6.14% (1.84–10.45)	77.63 ± 13.59	89.50 ± 6.82	17.44% (6.65–28.23)	0.20^d^

**Notes.**

a *n* = 6 participants.

b *n* = 7 participants.

c group × time effect.

d time effect.

## Discussion

The preliminary results from this pilot study suggest that a LVHIIT program consisting of short, manageable sessions is associated with improvements in selected health outcomes and a reduction in CVD risk factors in cancer survivors. Greater benefits were observed in the LVHIIT group despite the short time commitment per week. Improvements were also seen in the CLMIT group. The current study extends the research in the area of HIIT and introduces an additional clinical population by presenting preliminary evidence that cancer survivors may benefit from and participate safely in a low-volume form of high-intensity interval training (Aamot et al., 2014; Mattlage et al., 2013; Gillen et al., 2012; Gibala et al., 2012; Brito et al., 2014; Adamsen et al., 2009; Gist et al., 2014; Metcalfe et al., 2012). One of the most highly reported barriers to exercise adherence in cancer survivors is lack of time (Courneya et al., 2005; Ottenbacher et al., 2011). To improve adherence and increase participation in exercise, it could be argued that we require a more comprehensive understanding of what the minimum volume of exercise is in order for participants to experience the physiological and psychological benefits. To the best of the authors’ knowledge, there have been no studies reporting the effects of LVHIIT versus CLMIT on cancer survivors to date.

Improvements in QoL were seen, and in addition the LVHIIT group demonstrated improvements in two of the subscale domains (emotional wellbeing and functional wellbeing, Fig. 3). The improvements seen in emotional wellbeing in this group may be due to the increased physiological effect of the training protocol. Participants in this group anecdotally reported an increased level of enjoyment. The increased improvements seen in functional wellbeing in the LVHIIT group when compared with the CLMIT group (Fig. 2) may also be due to the increased physiological demand on working muscles in this training protocol. To date there is limited published data on the effects of CLMIT versus LVHIIT on QoL in cancer survivors, making it challenging to compare the differences seen in this study with other published work. A large Cochrane systematic review of exercise interventions on Qol in cancer survivors with 4,826 participants was carried out by Mishra et al. (2012). This review reported positive effects in QoL on those who participated in exercise, with more pronounced changes reported seen in those who participated in moderate or vigorous intensity exercise, when compared with participation in low intensity exercise. This review Mishra et al. (2012) grouped the both CLMIT and HIIT together making it difficult to compare the differences between the two protocols. Recently, positive changes in QoL have been reported in studies carried out by  Fong et al. (2012), Craft et al. (2012) and Ballard-Barbash et al., (2012). All three studies concluded that exercise has positive effects on psychological outcomes in cancer survivors, but did not report on the optimal exercise intensity to achieve these effects. Heinrich et al. (2015) found that by participating in a high-intensity functional training program, QoL improved significantly. In this study participants carried out crossfit type high-intensity resistance training over a five week period. The program included 15 sessions of high-intensity functional exercise and reported an increase of 6.3% in the emotional functioning of the participants using the European Organisational for Research and Treatment of Cancer (EORTC) core 30-item questionnaire (QLQ-C30) (Aaronson et al., 1993).

It was surprising to note that despite the low-volume of exercise, the groups showed significant reductions in anthropometric measures; hip circumference and waist circumference. The weight reductions showed a small effect size in both groups. Hip and waist circumference reductions demonstrated a small and medium effect size, respectively (see Table 1). Exercise induced weight loss, generally requires individuals to exercise at a moderate intensity for more than 150 min per week (Donnelly et al., 2009). Recent research on HIIT has shown reductions in anthropometric measures occurring at a much faster rate with less time overall (Aaronson et al., 1993). The reductions seen in the LVHIIT group may have been due to the positive effect on excess post-oxygen consumption which induces a larger post exercise energy expenditure (Larsen et al., 2014). An alternative and perhaps more plausible explanation is that diet was not controlled for, and as such the favourable changes in anthropometrical variables may have been as a result of participants altering their diets. The anthropometric changes seen in this study have been demonstrated in other clinical populations, but have not been reported in cancer survivors. A HIIT study in young obese females (Racil et al., 2013) reported a reduction in body mass, body fat percentage and BMI Z-score. The women participated in either a HIIT program or a CMIT program and participated in 12 weeks of exercise three times per week, the HIIT group achieved superior results in a number of variables which included; cholesterol, adiponectin, waist circumference and insulin resistance, when compared with the CMIT group (Racil et al., 2013). There seems to be lack of conclusive evidence on which intensity of exercise is more favourable for weight loss, both HIIT and CMIT have been shown to be useful in the obese population, but for cancer survivors there is limited evidence (De Feo, 2013). Tjonna et al. (2008) conducted a study in patients with metabolic syndrome who participated in either HIIT or CMIT three times per week over a 16 week period and found that both HIIT and CMIT were effective in reducing body weight and body fat. Overall HIIT demonstrated a superior effect on reducing risk factors for metabolic syndrome in the study by Tjonna et al. (2008). Recently Heinrich et al. (2015) showed significant decreases in fat mass and body fat percentage and an increase in lean mass after a 15 session, five week high-intensity functional exercise program in cancer survivors. Unlike the study by Heinrich, lean mass in the current study showed no changes in both groups. A five to 10% decrease in body fat can significantly reduce cardiovascular disease risk in overweight or obese individuals with type 2 diabetes (Wing et al., 2011), results from the present study demonstrates that the HIIT group reduced their fat mass by 4.97%.

HIIT has recently been promoted as an effective and time-efficient form of exercise for improving cardiovascular health (Gibala et al., 2012; Earnest, 2009; Jung et al., 2015). PWV has been shown to be one of the strongest measures of cardiovascular and all-cause mortality (Ramos et al., 2015; Vlachopoulos, Aznaouridis & Stefanadis, 2010). A study published in 2015 showed a decrease in PWV after participation in a cardiac rehabilitation program. The participants who carried out over 80% of the exercise sessions showed the positive results in PWV in this program. The exercise sessions were run three times per week over an eight week period and consisted of aerobic exercise at 70–85% of maximal heart rate (Oliveira et al., 2015). In the current study it appears as if CLMIT had a positive impact on PWV. Arterial stiffness increased in the LVHIIT group. The mechanism that underpins this finding is not fully understood, however, it may have been due to changes in the elastic properties in the arterial wall, both structurally and functionally. Although structural changes which modulate smooth muscle tone are likely to take longer than 12 weeks, an increase in sympathetic nervous system activity and an increase in concentrations of the vasoconstrictor hormones, could have elicited the change. Research has also found that HIIT is beneficial in reducing SBP and reducing arterial stiffness (Guimaraes et al., 2010; Racil et al., 2013). In the current study changes were seen in MAP, CSP, and CDP with a medium ES and a small ES was observed for AP and Aix (see Table 1). Vogel et al. (2013) and Heydari, Boutcher & Boutcher (2013) have both demonstrated similar findings in healthy individuals. There is evidence to support the current findings in other clinical populations, including stroke and hypertension (Mattlage et al., 2013; Guimaraes et al., 2010), but there is little evidence of the impact of LVHIIT on arterial stiffness and cardiovascular health in cancer survivors.

Reductions in insulin have been linked to a reduced risk of cancer reoccurrence and CVD, therefore improving health outcomes for participants. Elevated levels of insulin have been directly related to cancer risk and cancer recurrence (Giovannucci, 1995; Gallagher & LeRoith, 2011; Gregory et al., 2001). Participation in a 12 week home exercise program, developed for colorectal cancer survivors showed a reduction in insulin levels (Lee et al., 2013). The main outcome measure for this study was to increase participant’s physical activity levels by implementing a supported or unsupported exercise program consisting of brisk walking, hiking, stationary bike, swimming or aerobic exercise. Intensity was not controlled for. The program that provided support consisted of sessions with a trainer, education sessions and a DVD of exercise sessions. The unsupported program group was provided with written information, an exercise log book and pedometers, all of which were also given to the supported group. Both groups increased physical activity levels over the 12 weeks, and showed significant decreases in insulin levels (Lee et al., 2013). Currently it is recommended that the general population participate in moderate intensity exercise on most days of the week to improve insulin sensitivity (Mayer-Davis et al., 1998), but the optimal volume and intensity of exercise needed to improve insulin sensitivity in cancer survivors still needs to be determined. Although changes were not seen in the current study it is worth investigating further whether or not CLMIT and LVHIIT exercise programs have an impact on insulin levels in cancer survivors.

An improvement in the 6MWT test was evident in this study with a medium ES (see Table 1). Greater improvements were evident in the LVHIIT group in the 6MWT. The improvements in fitness observed in the current study have been seen in prior HIIT studies in both healthy individuals and participants with cardiometabolic disease (Gillen & Gibala, 2013). Changes were also seen in the STS test with a small effect size. Adamsen (2009) reported that low and high-intensity exercise during chemotherapy improved functional capacity in cancer survivors. Participants in the high-intensity group were prescribed a six week program which included a 30 min warm up, 45 min resistance training and 15 min of cardiovascular training (90 min sessions, six times per week) (Adamsen et al., 2009). More recently Heinrich et al. (2015) reported an improvement in the chair stand test and six minute walk test, after a five week high-intensity functional training program (60 min sessions, three times per week) in cancer survivors. Weston, Wisloff & Coombes (2014) reviewed 10 studies with 273 participants and looked at the effects of HIIT in people with cardiometabolic disease. This review reported on HIIT protocols of longer duration, two out of the 10 studies reviewed used a similar protocol to the current study, which included 30s for the interval and 60s for the recovery. Participation in the high-intensity protocol significantly increased cardiorespiratory fitness by almost double that of the moderate-intensity group (Weston, Wisloff & Coombes, 2014).

In summary, preliminary findings from this study suggest that both CLMIT and LVHIIT impart favourable benefits for cancer survivors with some greater improvements observed in the LVHIIT group, a medium ES was seen in a number of the reported variables. The changes may be due to the novel training stimulus carried out by the LVHIIT group, and there is a possibility that the CLMIT groups’ stimulus may be consistent with their usual activity levels. More importantly, there are generalised assumptions that HIIT is not achievable for those in some clinical populations and that vigorous activity may be harmful to those who have had a cancer diagnosis, but there are currently no findings to support these perceptions in the cancer population. Levinger et al. (2015) published a review in 2015 suggesting that the rate of adverse responses in a HIIT protocol is higher than in a moderate-intensity protocol, in those participants with cardiovascular and metabolic diseases. It also highlights that there is little evidence reported in this area, with only 156 individuals reviewed, it was suggested that more research should be conducted to provide further evidence on the efficacy of HIIT (Levinger et al., 2015). No adverse effects were reported by participants during this study. Caution was taken by providing supervision and close monitoring of the participants during and after the exercise sessions. An obvious limitation of the pilot study is the low number of participants, lack of a control group and no control over dietary intake. Our results provide an initial indication that LVHIIT may be a safe and time efficient mode of exercise to improve health outcomes and reduce CVD risk in cancer survivors. It appears that LVHIIT has a superior training effect in improving fitness levels, although it is unclear from these results if it is a superior training modality, when compared with CLMIT. A larger sample size with a control group is necessary to strengthen and validate the current findings.

##  Supplemental Information

10.7717/peerj.2613/supp-1 Data S1 Pre and post variables from 16 participants

## Additional Information and Declarations

### Data Availability

The following information was supplied regarding data availability: The data is available in Data S1. The authors request that you contact the corresponding author, Kellie L. Toohey (Kellie.Toohey@canberra.edu.au), when you download the raw data to facilitate useful discussions.
